# Topographical disorientation after ischemic mini infarct in the dorsal hippocampus: whispers in silence

**DOI:** 10.3389/fnbeh.2014.00261

**Published:** 2014-08-04

**Authors:** Jamshid Faraji, Nabiollah Soltanpour, Reza Moeeini, Shabnam Roudaki, Nasrin Soltanpour, Ali-Akbar Abdollahi, Gerlinde A.S. Metz

**Affiliations:** ^1^Department of Neuroscience, Canadian Centre for Behavioural Neuroscience (CCBN), University of LethbridgeLethbridge, AB, Canada; ^2^Faculty of Nursing and Midwifery, Golestan University of Medical SciencesGorgan, Iran; ^3^Department of Anatomy, Biology and Molecular Research Center, Babol University of Medical SciencesBabol, Iran; ^4^Department of Behavioural Studies, Avicenna Institute of NeuroscienceYazd, Iran

**Keywords:** silent stroke, endotelin-1, hippocampus, ziggurat task, return behavior, spatial navigation, topographical disorientation, early cognitive decline

## Abstract

Silent focal ischemic mini infarcts in the brain are thought to cause no clinically overt symptoms. Some populations of hippocampal cells are particularly sensitive to ischemic events, however, rendering hippocampal functions especially vulnerable to ischemia-induced deficits. The present study investigated whether an otherwise silent ischemic mini infarct in the hippocampus (HPC) can produce impairments in spatial performance in rats. Spatial performance was assessed in the ziggurat task (ZT) using a 10-trial spatial learning protocol for 4 days prior to undergoing hippocampal ischemic lesion or sham surgery. Hippocampal silent ischemia was induced by infusion of endothelin-1 (ET-1), a potent vasoconstrictor, into either the dorsal or the ventral hippocampus (dHPC and vHPC). When tested postoperatively in the ZT using a standard testing protocol for 8 days, rats with hippocampal lesions exhibited no spatial deficit. Although spatial learning and memory in the ZT were not affected by the ET-1-induced silent ischemia, rats with dHPC stroke showed more returns when navigating the ZT as opposed to the vHPC rats. Comparison of region-specific HPC lesions in the present study indicated that dorsal hippocampal function is critically required for topographic orientation in a complex environment. Topographic disorientation as reflected by enhanced return behaviors may represent one of the earliest predictors of cognitive decline after silent ischemic insult that may be potentially traced with sensitive clinical examination in humans.

## Introduction

Silent brain infarctions are silent radiologic abnormalities without overt stroke-like functional symptoms (Vermeer et al., 2007; Kim et al., 2011). Although most silent infarcts initially have no lasting impact on daily life activities, they indicate a greater risk of transient ischemic attacks or major stroke and therefore are of particular clinical interest (Herderscheê et al., 1992; Vermeer et al., 2003; Kim et al., 2011). The cumulative damage caused by multiple silent strokes also may play a key role in the pathogenesis of cognitive and neurobehavioral disturbances (Herderscheê et al., 1992; Masuda et al., 2001; Vermeer et al., 2003; Kim et al., 2011). Despite the lack of overt stroke-like symptoms in silent strokes, it is believed that they are associated with subtle deficits in cognitive function that typically remain unnoticed (Vermeer et al., 2007). Furthermore, the first silent stroke is often followed by gradual deterioration of cognitive function and hippocampal shrinkage (Lopez et al., 2003; Vermeer et al., 2003; Blum et al., 2012). Thus, the hippocampus (HPC) represents a particularly relevant target for pre-clinical studies in silent stroke.

It seems reasonable to expect that the HPC is sensitive to silent ischemic events (Driscoll et al., 2008). Their dense innervation patterns, excitotoxicity and increased calcium entry rates renders CA1 pyramidal cells and hilar interneurons at a relatively greater risk to ischemia-induced neuronal damage compared to the CA3, denate gyrus or other structures (Johansen et al., 1987; Hsu and Buzsáki, 1993). Work in animal models, using endothelin-1 (ET-1), an endogenous potent and long-acting vasoconstricting peptide (Yanagisawa et al., 1988), showed that multiple ET-1 injections into the HPC (Spanswick et al., 2009; Faraji et al., 2011a) are associated with significant and permanent loss of hippocampal tissue and a robust spatial impairment. The HPC is centrally involved in goal-directed spatial navigation (Morris et al., 1982; Sutherland et al., 1982; Astur et al., 2002; Ergorul and Eichenbaum, 2004; de Hoz et al., 2005; Faraji et al., 2008). However, a regionalized process or functional specialization in different parts (left vs. right and dorsal vs. ventral) of the HPC indicates that the HPC may not act as a functionally unitary structure (Moser and Moser, 1998). In rodents, hippocampal function in the dorsal (septal pole) region differs relative to its ventral (temporal pole) area so that the former mediates learning and memory and the latter anxiety-related emotional behaviors (Bast and Feldon, 2003; Bannerman et al., 2004; Wang et al., 2013). It is important to note that most evidence on the functional segregation between the dorsal and ventral hippocampus (dHPC and vHPC) arises from studies using a water maze task (WT), a wet-land task that imposes a stressful situation and relies on aversive motivation (D’Hooge and De Deyn, 2001; Aguilar-Valles et al., 2005).

The differential role of dHPC and vHPC in spatial navigation suggests that the HPC represents an ideal structure to investigate the symptomatic and structural consequences of hippocampal silent ischemic mini infarcts. Here, we induced silent ischemic mini infarcts by injection of a miniscule ET-1 concentration (Driscoll et al., 2008) into the dHPC and vHPC in rats. Animals were tested in spatial navigation using the ziggurat task (ZT), a dry-land maze for spatial cognition which employs appetitive motivation. The ZT has demonstrated particular sensitivity to spatial cognitive deficits after cerebral infarcts (Faraji et al., 2009). We hypothesized that in spite of the silent nature of the ischemic lesion used in the present study, dorsal and ventral lesions to the hippocampus may produce different behavioral profiles in the distinct procedural and route system of the ZT. The findings provide new insights into distinct behavioral profiles of dHPC vs. vHPC. Our new rat paradigm may be the clinically relevant model for the mechanisms of gradual hippocampal cell loss and cognitive impairments in silent stroke.

## Materials and method

### Animals

Twenty male Wistar rats (4 months of age) were housed in pairs at 20 ± 1°C and kept on a 12-h light/dark cycle (light on from 07:00 to 19:00). Animals were randomly subdivided into three groups: dorsal hippocampus (dHPC) lesion, *N* = 7; ventral HPC (vHPC) lesion, *N* = 7; Sham, *N* = 6. Prior to the testing rats were handled for approximately 4–5 min daily for four consecutive days. Animals were tested in the ZT for 4 days pre-lesion, and for 8 days post-lesion. Animals received water *ad libitum* and a restricted diet starting 4 days prior to training and testing in the ZT. Animals were weighed daily and body weight was maintained at about 85% of their initial body weight by providing additional amounts of food in their home cage at least 2–3 h after completion of behavioral testing. All procedures were approved by the Avicenna Institute of Neuroscience (AIN) Animal Care Committee and were carried out in accordance with NIH guidelines.

### Behavioral training and testing

#### Ziggurat task (ZT)

**Figure 1:**
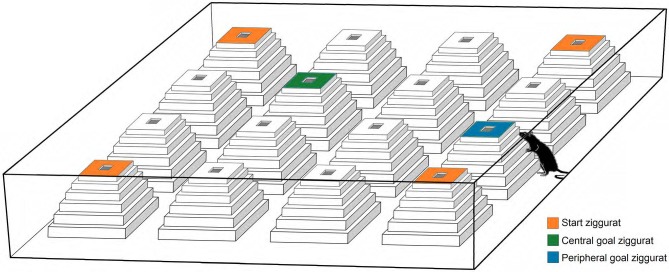
**A schematic representation of the Ziggurat task (ZT)**. The task consisted of an open-field box constructed from opaque white laminate and 16 ziggurats, and required rats to learn and remember that the top surface of one of 16 ziggurats in the open field is baited with a food reward. All ziggurats were identical and made of white styrofoam covered by transparent duct tape. The standard or non-cued version of the ZT used in the present study requires animals to use spatial (distal and/or proximal) cues to navigate to the goal ziggurat. On testing days, the rats are released from each starting point (orange ziggurats) and explore the environment in which only one goal ziggurat (peripheral or central; blue and green ziggurats, respectively) is baited (Faraji et al., 2008).

All procedures for ZT testing were similar to previously reported (Faraji et al., 2008, 2010, 2011a). The ZT environment is illustrated in Figure 1. Prior to behavioral testing, rats were habituated to the ZT environment for 10 min each day for 4 days. In ZT environment animals must use spatial cues (distal and/or proximal) to navigate to the goal ziggurat.

#### Pre-ischemic spatial performance

Immediately following the last session of habituation, the testing sessions were conducted over 10 trials per day for four consecutive days within the standard version of the ZT. The cycle consisted of alternating *different-goal* or learning days (days 1 and 3) and *same-goal* or memory days (days 2 and 4). On the learning days, the goal ziggurats representing stepped pyramids (either peripheral or central) were located in a new location, and rats were required to find and learn the location of the goal ziggurat in the new place. The goal ziggurats remained in the same place on the memory days. Thus, the rats were required to remember the location they had learned previously. Two sets of ziggurats were defined in the arena. First, “start” ziggurats, located in each corner, and second, the rest of ziggurats or “goal” ziggurats. On the testing days, the rats, released from each starting point, were allowed to explore the environment. One peripheral goal ziggurat was baited with spaghetti for each trial on days 1 and 2. On days 3 and 4, however, rats were rewarded on the central goal ziggurat. During each testing day, rats explored the environment in 10 trials each and from four different starting points at a randomized position. Across trials, the starting location varied among the four corners of the apparatus, and on each trial, animals navigated in the environment for 80 s or until they found the goal ziggurat. Since the location of the goal ziggurat remained constant from trial to trial for every 2 days, the animals had to learn and remember the new locations of the goal ziggurat for every 2 days.

#### Post-ischemic spatial performance

The procedures for spatial performance assessment after the HPC ischemia were identical to those described in pre-ischemic sessions, except that the testing days were increased to 8 days. Rats were tested in the ZT for 4 days (days 1–4) with peripheral goal ziggurats and for four additional days (days 5–8) with central goal ziggurats. Using peripheral-central ziggurats or mixed procedure (Faraji et al., 2008) in the present experiment, it was assumed that rats needed to change the peripheral strategies to central strategies every 4 days.

**Figure 2:**
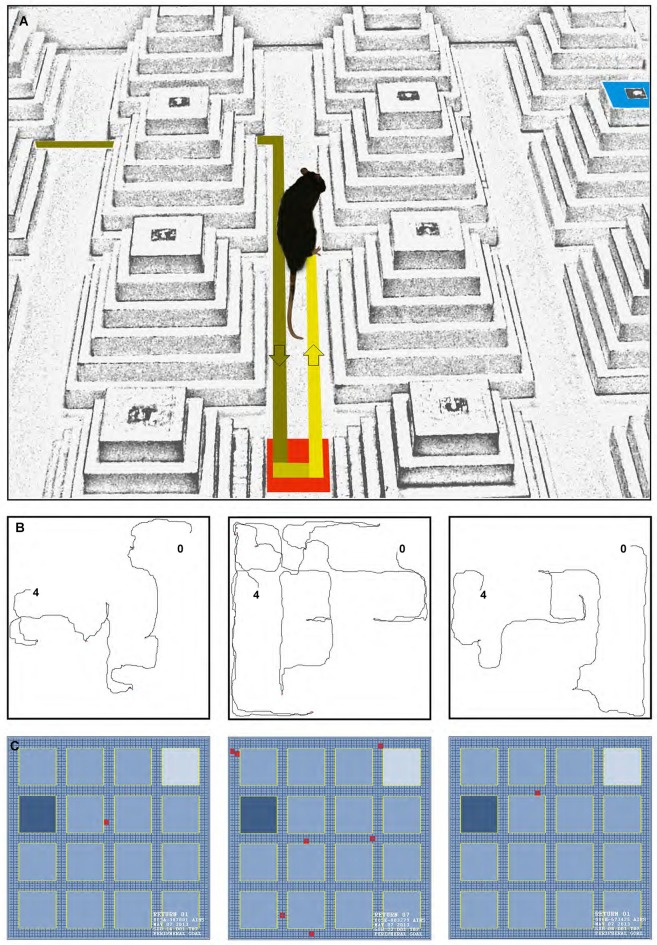
**(A)** A photograph of a return that can typically occur during the spatial navigation in the ziggurat task. (**B**, left to right) Path trajectories recorded on Trial seven on the learning day by a control, dHPC, and vHPC rat from starting point (0) to the peripheral goal ziggurat (4). Note the increased number of returns taken by the dHPC rat (middle sub-panel) during the goal-directed navigation in the ZT. **(C)** A motiongraph of the number of returns taken by the same rats. Light and dark blue squares represent start and goal ziggurats, respectively and red squares indicate the number of returns calculated by the software (SINA motiongraph, V.II, 2011).

In addition to latency (time spent to find either of the peripheral or central goals), path speed (calculated by dividing the path length by the latency), path length, and returns were evaluated. Returns (Figure 2A) were characterized by the different pathways animals chose to return to the goal ziggurat in order to accomplish the task. Returns typically refer to the act of localizing and going back to the goal ziggurat during the goal-directed navigation (Faraji et al., 2008; Figure 2B). The movements of the animals were recorded and analyzed by HVS Image tracking system. Furthermore, returns were analyzed and tracked by a motiongraph software (SINA motiongraph, V.II, 2011, Tabriz, Iran; Figure 2C). A return was characterized by one stop (i.e., speed of 0.0 m/s lasting at least 1 s) followed by creation of a 180° angle towards left or right on the current route.

### Ischemic lesion procedure

Fourteen rats in two groups (dHPC, *N* = 7; vHPC, *N* = 7) received one unilateral infusion of a low concentration of ET-1 (7.5 pmol; Sigma-Aldrich, USA; 0.5 μL in saline solution; 0.1 μL/min; McDonald et al., 2008; Faraji et al., 2009) to the dHPC and vHPC through a 23-gauge cannula attached to a Harvard infusion pump-11 Plus (USA). The concentration of 7.5 pmol has been shown to cause significant structural damage in the HPC without any functional impairment in the Morris water task (MWT; Driscoll et al., 2008). Stereotaxic coordinates for the ET-1 infusion into HPC were based on the Paxinos and Watson (1997) atlas: AP: −4.16; ML: ±3.4; DV: −3.2 (dHPC) and AP: −5.60; ML: ±5.20; DV: −5.20 (vHPC) in millimeters relative to the bregma-lambda distance. Left or right HPC for ET-1 infusion in each rat was assigned in a pseudorandom order. The cannulae were left in place for 5 min after infusion to allow for diffusion of ET-1 from the tip. The scalp was sutured after surgery and the animal was given analgesic (buprenorphine HCl, 0.05 mg/kg, s.c.), and antibiotic (Terramycine, 60 mg/kg, i.m.). The Sham group (*N* = 6) received all surgical procedures and postoperative treatments except skull trephination and infusion. Skull trephination was not performed in sham-operated animals because it has been previously reported to produce behavioral and neurochemical asymmetries (Adams et al., 1994). Animals were allowed to recover for 3–4 days before the beginning of the post-lesion spatial testing.

### Histological evaluation of lesion extent and location

Rats were euthanized with an overdose of sodium pentobarbital (300 mg/kg i.p.) and intracardially perfused with saline (0.9%; 200 mL/rat) followed by 4% paraformaldehyde (PFA; 200 mL/rat). Brains were removed, post-fixed for 24 h in 4% PFA and cryoprotected in 30% sucrose and 4% PFA at 4°C for coronal sectioning (40 μm) and cresyl-violet staining. The stained sections were examined under a microscope (Nikon, Japan) and images were captured using an AxioCam camera (Carl Zeiss, Germany) for histological analysis and presentation. In order to measure the extent of HPC damage in each lesion rat, four images from dHPC and vHPC were captured under 1× magnification, corresponding approximately to −3.60, −3.80, −4.16, and −4.30 (dHPC) and −5.30, −5.60, −5.80 and −6.40 (vHPC) mm relative to bregma. A systematic sampling grid with an area per point of 20,000 pixels was randomly projected on each image and the number of points hitting the intact HPC region was counted. Grids were generated using ImageJ software (NIH, USA). The total number of hits in each rat was then divided by the average number of hits obtained by three control rats. The complement proportion was used as the percentage HPC (dorsal or ventral) lesion estimate (Faraji et al., 2011a).

### Data analysis

The results were subject to analysis of variance (ANOVA) for repeated measures across testing sessions, dependent and independent *t*-tests. Four spatial behavioral indices within the ZT (i.e., latency, path speed, path length and the number of returns) were averaged for 10 trials and analyzed for each learning and memory day. Repeated measures ANOVA was conducted with group (Sham, dHPC, vHPC), day (days 1–8) and trial (trials 1–10) as independent measures. Latency, path speed, path length and the number of returns served as dependent variables. A *post hoc* test was used to adjust for multiple comparisons. For all histological data the differences in between-group and within-group comparisons were assessed with dependent and independent sample *t*-tests. Correlation coefficients were calculated to examine the correlation between lesion extent and spatial performance on ZT. Familywise error rate was considered prior to the multiple *post hoc* analyses if necessary. In order to avoid familywise error or type I error inflation, and prior to any further *post hoc* analysis (e.g., dependent and independent *t*-tests) on the same set of the data, familywise error followed by the Bonferroni correction (α_*B*_) for multiple comparisons was estimated. Significance level used for all tests was 0.05, and is denoted by an asterisk in all graphs. Data are presented as means ± SEM.

## Results

### Histology and lesion extent

**Figure 3:**
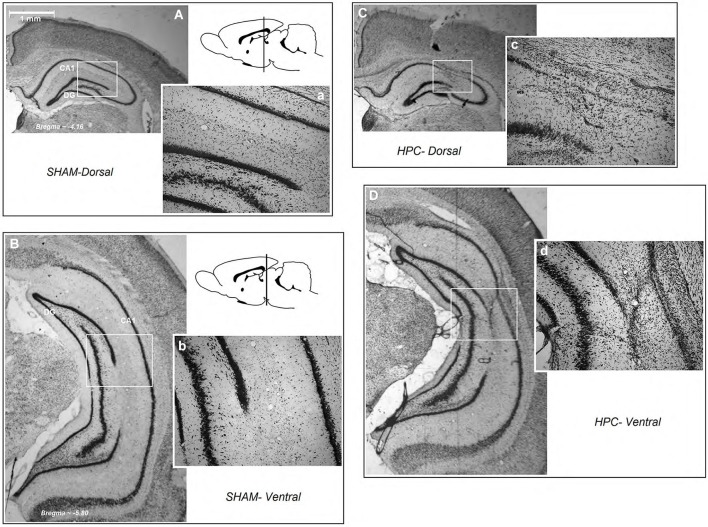
**Schematic illustration of dorsal and ventral hippocampus lesions.** (panels **Aa** and **Bb**) Photomicrograph of a coronal section of an intact dorsal and ventral region of the hippocampus (**A** and **B**; magnification 1×, and **a** and **b**; magnification 10×). **(Cc)** A dHPC and **(Dd)** a vHPC rat. Both low and higher magnifications of the dorsal and ventral hippocampus show that all ischemic rats displayed similar tissue loss resulting from ET-1 infusion. HPC-D: dorsal hippocampus; HPC-V: ventral hippocampus.

**Figure 4:**
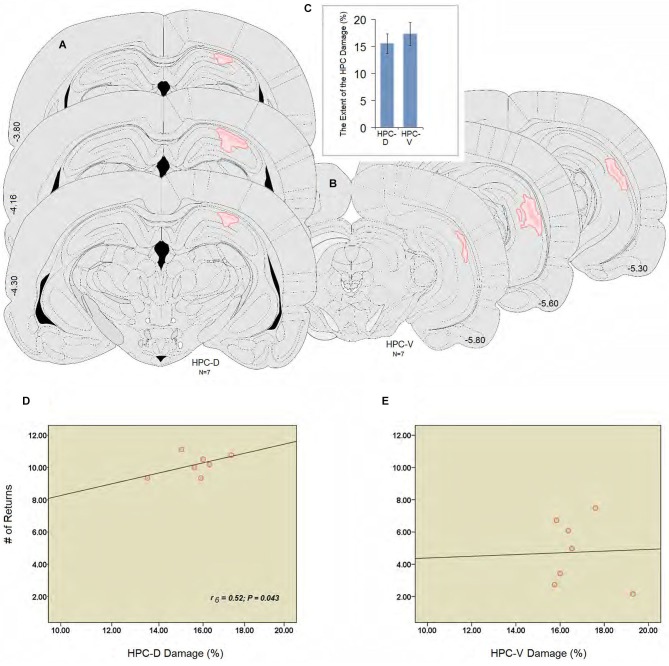
**(A and B)** Illustrations of the lesion observed unilaterally through the rostral and caudal extent of the hippocampus (dHPC: ~−3.80, −4.16, −4.30; vHPC: ~−5.30, −5.60, −5.80). The dark pink area represents the lesion core which is surrounded by the light pink area or penumbral zone with less tissue damage in the dorsal and ventral hippocampus. **(C)** Estimate of the damage percentage for the dHPC and vHPC groups in the dorsal and ventral hippocampus. Both groups were equally affected by the ET-1 infusion into the hippocampus. (**D** and **E**) Correlation between the percent damage induced by ET-1 in the dorsal **(D)** and the ventral **(E)** hippocampus, and the number of returns during spatial investigation in the ZT. The number of returns was significantly correlated to the damage percent only in the dHPC rats. Error bars show ±SEM. Atlas plates are from Paxinos and Watson (1997). HPC-D: dorsal hippocampus; HPC-V: ventral hippocampus.

Figure 3 illustrates an intact dHPC and vHPC (Aa & Bb) from a sham animal compared to the extent of an ischemic lesion induced by ET-1 in the dHPC (Cc) and vHPC (Dd) animals. There were no signs of tissue damage in the HPC of any of the animals in the sham group. The HPC lesion in both groups was mainly restricted to the region in and around the infusion sites. HPC ischemia in the dHPC was mostly limited to the CA1 and some portions of the CA3. Cell bodies in affected regions seemed ablated and the pyramidal cell layer appeared faint and indistinct. Evidence of ischemic damage to the CA2 and dentate gyrus (DG) was observed in one animal of the dHPC group. Extra-HPC damage in dHPC rats was very minor or nonexistent. In all vHPC animals, on the other hand, ET-1 infusion induced ischemic damage in the CA1 as well as detectable cell damage in ventral CA2 and CA3. Two animals in the vHPC also indicated superficial tissue loss in the primary auditory cortex (Au1) and secondary auditory cortex, ventral (AuV), but were still included in the statistical analysis. An independent *t*-test performed on the percentage of tissue volume loss in the dHPC and vHPC yielded no significant difference between groups (15.86 ± 1.47% vs. 17.29 ± 1.51%, respectively; *P* > 0.77; Figures 4A–C). These observations indicate that dHPC and vHPC groups were structurally affected by the ET-1 infusion and the extent was comparable between these groups.

Spearman’s rank correlation coefficients were calculated to investigate correlation between percent lesion size and spatial performance. There were no statistically significant correlations between latency, speed and length, and the lesion size either in dHPC or vHPC. Analysis of performance in relation to the ischemic insult in dorsal or ventral regions of the HPC and the number of returns, however, revealed a significant correlation between the lesion size only in the dHPC and the number of returns in the ZT (*r_6_* = 0.52; *P* = 0.043; Figures 4D,E).

### Pre-ischemic assessment of spatial performance

**Figure 5:**
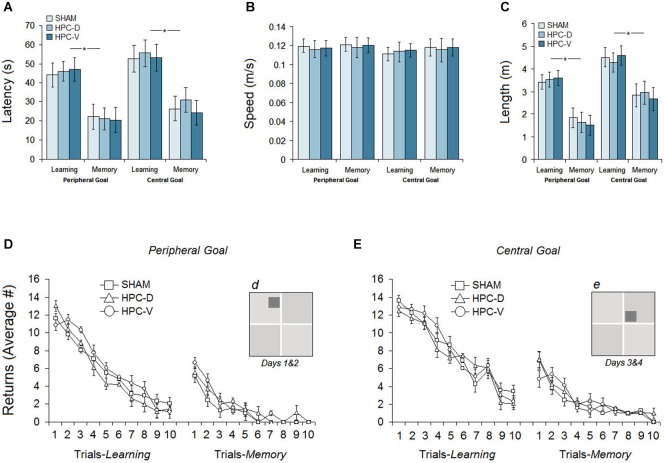
**Pre-ischemic spatial testing in the ZT**. **(A)** Average latency to locate the peripheral or central goal ziggurats during 4 days of spatial testing. All groups significantly spent less time on memory days to find the spatial goals compared to learning days. **(B)** Mean path speed averaged across 4 days of testing. **(C)** Mean path length (distance traveled) to locate the spatial goal during each day of testing (learning and memory) on peripheral- and central-goal trials. (**D** and **E**) The number of returns for three groups on learning and memory days during spatial navigation in the ZT to locate peripheral and central goal ziggurats. **(d and e)** Dark gray squares indicate the location of peripheral and central spatial goals, respectively.

The results of the pre-ischemic spatial testing in the ZT for 4 days are illustrated in Figures 5A–E. Spatial testing prior to infusion of the ET-1 in the HPC indicated no differences between groups in their latency (time spent to find the goal ziggurat), path speed, path length and returns.

#### Latency

The ability of the rats in each group to acquire the new spatial goal location (peripheral or central) on learning days (Sham: 48.39 ± 6.2 s, vHPC: 51.72 ± 6.6 s, vHPC: 49.33 ± 6.2 s) and to remember the location of the spatial goals on memory days (Sham: 23.08 ± 5.2 s, dHPC: 26.13 ± 5.6 s, vHPC: 22.49 ± 5.7 s) is averaged in Figure 5A. Repeated measure ANOVA showed no significant effect of Group (*P* > 0.49) but a significant effect of Day (*F*_(1,17)_ = 9.66, *P* < 0.038; *η*^2^ = 0.44) indicating that all groups spent less time to find the spatial goal ziggurat on memory days compared to learning days. No interaction between Group and Day was found. The observed differences between learning and memory days was also supported by independent *t*-test in each group (Sham: *t*_(10)_ = 9.56, *P* < 0.032; dHPC: *t*_(12)_ = 11.87; *P* < 0.026; vHPC: *t*_(12)_ = 13.01; *P* < 0.021).

#### Path speed

All three groups showed relatively constant speeds (Figure 5B) across the four testing days in the ZT (Learning: Sham 0.121 ± 0.008 m/s, dHPC: 0.118 ± 0.008 m/s, vHPC: 0.119 ± 0.007 m/s; Memory: Sham 0.123 ± 0.009 m/s, dHPC: 0.120 ± 0.011 m/s, vHPC: 0.122 ± 0.011 m/s). There was no significant difference between groups on learning (*P* > 0.66) and memory days (*P* > 0.72) in terms of path speed.

#### Path length

Although latency and path length (distance traveled) in both wet-land (e.g., MWT) and dry-land (e.g., ZT) arenas typically reveal similar profiles of spatial navigation (Vorhees et al., 2004; Faraji et al., 2010), we have reported the rats’ path length in the present experiment for further consideration. Examination of acquisition in terms of path length to locate the spatial goal ziggurat revealed that rats in all groups showed shorter path length (Figure 5C) during the spatial investigation in peripheral-goal compared to central-goal sessions (Learning: 3.51 ± 0.33 m vs. 4.46 ± 0.43; Memory: 1.67 ± 0.44 m vs. 2.82 ± 0.51) suggesting that spatial navigation to locate central goals was more difficult than navigation to locate peripheral goals. A significant effect of Day (learning vs. memory) was observed (*F*_(1,17)_ = 9.16, *P* < 0.037; *η*^2^ = 0.33) indicating that all rats were able to remember the spatial location of goal ziggurats. Neither the Group effect (*P* > 0.061) nor the Group by Day interaction (*P* > 0.69) were significant. The main effect of Location (peripheral goal vs. central goal), however, was significant (*F*_(1,16)_ = 11.07, *P* < 0.042; *η*^2^ = 0.31) suggesting that central goals on learning and memory days for all groups were less likely to be investigated than the peripheral goals.

#### Returns

Results showing the number of returns on learning and memory days for both peripheral and central-goal trials are depicted in Figures 5D,E. As expected, the number of returns on the first trials was quite high for all groups on both peripheral- and central-goal trials, reflecting spatial disorientation and inadequate experience in locating the goal ziggurats. All three groups, however, showed a similar amount of improvement indicated by decreased number of returns over the next trials on both learning and memory days (Learning: Sham 5.79 ± 0.65, dHPC 5.47 ± 0.65, vHPC 6.24 ± 0.66; Memory: Sham 1.15 ± 0.35, dHPC 1.65 ± 0.47, vHPC 1.79 ± 0.35). No significant effect of Group was found in terms of the number of returns in the task (*P* > 0.39). In addition, the profile of returns in all groups showed that although they improved the return-based investigation on the last trials of spatial testing in the same pattern, the number of returns in most rats during the central-goal trials (Learning: Sham 7.86 ± 0.69, dHPC 7.47 ± 0.67, vHPC 7.94 ± 0.68; Memory: Sham 2.17 ± 0.37, dHPC 2.58 ± 0.50, vHPC 2.22 ± 0.38) was higher compared to peripheral-goal trials. The observed difference, however, was not significant (*P* > 0.11).

In summary, pre-ischemic spatial measures in the ZT showed that all groups were able to learn and remember the location of the spatial goal ziggurats in a similar time course and manner.

### Post-ischemic assessment of spatial performance

**Figure 6:**
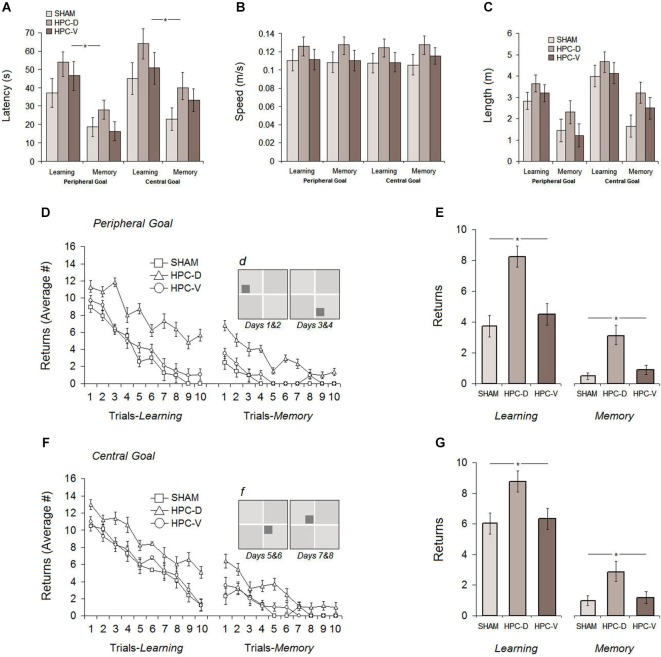
**Post-ischemic spatial testing in the ZT**. Average latency **(A)**, path speed **(B)** and path length **(C)** to find the spatial goal in the task showed comparable spatial performance among all experimental groups during 8 days of testing. Rats with silent ischemia in the dorsal hippocampus (dHPC), however, showed significantly increased number of returns either in the task with peripheral (**D** and **E**) or central goal (**F** and **G**) when compared to vHPC and sham rats on learning and memory days. Note that spatial testing on the first four post-ischemic days (learning and memory) used peripheral goal ziggurats and on the second four post-ischemic days used the central goal ziggurats. Dark gray squares in sub-panels **(d)** and **(f)** indicate the location of peripheral and central spatial goals, respectively. *P < 0.05; error bars show ± SEM. HPC-D: dorsal hippocampus; HPC-V: ventral hippocampus.

The procedure of spatial assessment after focal ischemic infarct in the dHPC and vHPC was identical to those used in the pre-ischemic phase with the exception that rats were required to navigate in the task for 8 days. A 4-day protocol for testing spatial performance with peripheral goal ziggurats followed by four additional days for testing with central goal ziggurats was employed. Figures 6A–G displays the results of the post-ischemic testing in the ZT.

#### Latency

Figure 6A shows the average latency to find the goal ziggurat in the task for all groups over 8 days of acquisition (Learning: Sham: 41.12 ± 5.9 s, dHPC: 59.07 ± 9.6 s, vHPC: 50.61 ± 8.3 s; Memory: Sham: 18.71 ± 5.3 s, dHPC: 33.09 ± 9.6 s, vHPC: 22.79 ± 9.7 s). Despite higher latency to find the goal ziggurats in the dHPC rats on both learning and memory days, repeated measure ANOVA conducted for ZT testing after ischemic lesion only revealed a significant effect of Day (*F*_(1,17)_ = 5.07, *P* < 0.042; *η*^2^ = 0.24) indicating that all groups were able to acquire and retrieve the location of the goal ziggurats in the same manner. A significant effect of Trial (*F*_9,17_ = 16.11, *P* < 0.038; *η*^2^ = 0.30) but no effects of Group (*P* > 0.083), Group by Day (*P* > 0.069) and Group by Trial (*P* > 0.18) was found. An additional dependent samples *t*-test conducted for learning and memory days in each group showed that all groups were able to remember the spatial location of goal ziggurats on memory days (Sham: *t*_10_ = 7.48, *P* < 0.032; dHPC: *t*_(12)_ = 5.09; *P* < 0.04; vHPC: *t*_(12)_ = 9.13; *P* < 0.036). Thus, post-ischemic latency in the ZT was not affected by the lesion induced by ET-1 in the dHPC and vHPC. This also suggests that ischemic animals performed comparably, regardless of the location of the infarct in the HPC.

#### Path speed

Because latency can be potentially affected by differences in path speed and, more importantly, ischemia-induced hyperactivity as previously reported (Plamondon et al., 2008) path speed, in addition to latency, was considered within the ZT. All groups displayed relatively constant speed (panel B) during spatial navigation in the task (Learning: Sham 0.114 ± 0.007 m/s, dHPC: 0.126 ± 0.008 m/s, vHPC: 0.117 ± 0.008 m/s; Memory: Sham 0.112 ± 0.007 m/s, dHPC: 0.129 ± 0.01 m/s, vHPC: 0.119 ± 0.01 m/s), although dHPC rats showed a slightly higher speed than other groups. ANOVA did not show a significant effect of Group (*P* > 0.14) and Day (*P* > 0.63) indicating that the procedure to induce ischemic insult in the HPC used in the current study had no major impact on the animals’ speed during spatial navigation in the ZT.

#### Path length

The distance traveled by rats in different groups within the ZT is depicted in panel C. As observed in the pre-ischemic assessment of spatial performance, all groups traveled a similar distance during navigation in the task, although they generally performed more travels during central-goal trials than peripheral-goal sessions (central-*learning*: 4.22 ± 0.49 m vs. peripheral-*learning*: 3.20 ± 0.40 m; central-*memory*: 2.43 ± 0.51 m vs. peripheral-*memory*: 1.64 ± 0.54 m). Furthermore, dHPC rats performed more travels across all testing days compared to other groups, but the observed difference was not significant (*P* > 0.061). Once again, all groups significantly indicated less travels on memory days when they were required to retrieve spatial information of the goal ziggurat in the task compared to learning days (*F*_(1,17)_ = 12.51, *P* < 0.039; *η*^2^ = 0.23). Furthermore, as observed in the pre-ischemic spatial testing, a significant effect of Location (central vs. peripheral goal) was also found (*F*_(1,17)_ = 9.28, *P* < 0.046; *η*^2^ = 0.21) in the post-ischemic distance traveled by all groups suggesting that spatial investigation or goal-directed navigation during central-goal trials for all rats was more difficult than peripheral-goal trials regardless of their experimental conditions. No significant interaction between Group and Day (*P* > 0.19), and Group and Location (*P* > 0.066) was observed.

#### Returns

The number of returns during post-ischemic spatial investigation in the task revealed different profiles of ischemia-induced behavioral alterations in rats with dHPC ischemic insult compared to vHPC rats. As can be seen in Figures 6D,F, all groups showed noticeable improvement in their spatial navigation indicated by the decreasing number of returns after ischemic insult in peripheral- and central-goal sessions. dHPC rats, however, engaged in a larger number of returns during spatial navigation in the task when compared with other groups. ANOVA applied to the post-ischemic number of returns yielded significant effects of Group (*F*_(2,17)_ = 10.03, *P* < 0.026; *η*^2^ = 0.38) and Day (*F*_(7,16)_ = 6.11, *P* < 0.042; *η*^2^ = 0.28) but no Group by Day interaction (*P* > 0.17). *Post hoc* testing (Tukey HSD) revealed a significant difference between dHPC group and sham (*P* < 0.028) as well as between dHPC and vHPC rats (*P* < 0.031). No difference was observed between sham and vHPC groups in terms of the number of returns (*P* > 0.16). It is noteworthy that the dHPC group did not show return-free spatial investigation over the 80 (10 trials × 8 days) trials of post-ischemic spatial testing. Moreover, although all groups were able to learn and remember the spatial location of goal ziggurats, an additional between-group comparison showed that dHPC rats had significantly more returns in the task on both learning (different-goal) and memory (same-goal) days (all *P*s < 0.05; ANOVA) indicating that spatial navigation within the ZT was affected by silent ischemic insult in dorsal, but not ventral HPC. No significant effect of Location, on the other hand, was found (*P* > 0.069) suggesting that the number of returns performed by all groups in the peripheral-goal trials (Learning: Sham 3.73 ± 0.46, dHPC 8.25 ± 0.44, vHPC 4.49 ± 0.45; Memory: Sham 0.49 ± 0.25, dHPC 3.08 ± 0.27, vHPC 0.90 ± 0.24) and central-goal trials (Learning: Sham 6.03 ± 0.53, dHPC 8.76 ± 0.56, vHPC 6.34 ± 0.56; Memory: Sham 0.99 ± 0.15, dHPC 2.89 ± 0.23, vHPC 1.19 ± 0.15) was equal.

Overall, post-ischemic assessment of spatial performance indicated that inducing silent ischemia by infusion of ET-1 in the dHPC and vHPC did not produce any detectible effect on spatial learning and memory measured by latency, path speed and path length in the ZT. All rats were able to acquire and retrieve the spatial information in similar rate regardless of their experimental situation. However, dHPC rats exclusively exhibited a considerable number of returns during the goal-directed investigation or spatial navigation in the ZT.

## Discussion

Results in the present study show that in the presence of permanent neuronal damage in the dHPC and vHPC, focal ischemic ET-1 lesions did not alter spatial learning and memory measured by latency, speed and length in the ZT. Thus, the ET-1 lesions resemble the clinical features of a silent stroke. Nevertheless, dorsal compared to ventral hippocampal lesions result in different strategies indicated by the enhanced return behaviors during spatial investigation. Therefore, this behavior may represent one of the most sensitive and distinctive indicators of dorsal hippocampal function.

### Silent stroke may not always be silent: reasons for whispers in silence

Asymptomatic cerebrovascular lesions or silent strokes are typically defined as cerebrovascular pathological conditions that can result in tissue damage in the absence of noticeable functional consequences or stroke-like symptoms (Kim et al., 2011). Symptoms of silent stroke, therefore, may go unreported in spite of lasting tissue damage. Considering the particular vulnerability of regions of the HPC to ischemic events (Johansen et al., 1987; Schmidt-Kastner and Freund, 1991; Hsu and Buzsáki, 1993), the association of smaller hippocampi and memory decline in silent stroke (Blum et al., 2012), the HPC represents a clinically relevant target for pre-clinical studies in silent stroke. Intracerebral infusion of ET-1 may represent a particularly suitable method due to its properties to reduce local blood flow and produce ischemic lesions in a dose-dependent manner (Yanagisawa et al., 1988). Ischemic lesions induced by the intraparenchymal infusion of ET-1 are likely caused by reduced blood flow in the target region rather than by direct action of ET-1 on brain tissue (Konopková et al., 2012). Moreover, because blood flow reduction following the ET-1 infusion into the brain tissue is rapid, but not immediate (Macrae et al., 1993) and reperfusion happens over several hours (Biernaskie et al., 2001), the ischemic lesion occurring in the cerebral tissue may be more representative of human stroke than the immediate reduction and reperfusion seen in other animal models of ischemic stroke (Faraji et al., 2011b).

Because some populations of hippocampal cells are highly sensitive to ischemic events (Sachdev et al., 2007), we infused low-concentration ET-1 into the HPC as a model of localized subthreshold stroke (Driscoll et al., 2008; McDonald et al., 2008; Faraji et al., 2009). The present morphological assessments indicate that ET-1 was able to induce ischemic loss of tissue that focally occurred in both dorsal and ventral CA1 subregions. In the present study, ischemia localized in the dHPC was not able to induce spatial impairment in the ZT due to a minor extent of tissue loss. While the average lesion size in our study was ~16% for the dorsal region, it has been previously reported that spatial impairment required further extent of lesion in this region to induce noticeable impairments (Moser et al., 1995). Nevertheless, because a tendency towards increased latency and path length was observed in the dHPC rats, further studies with larger group size may assist in confirming the results of the present study.

In spite of significant and permanent hippocampal tissue damage, the spatial standard measures of goal-directed navigation within the ZT revealed no impairments in cognitive functions. These observations are consistent with previous reports in which that despite damaged neuronal tissue in the HPC, there was no obvious cognitive impairments after transient vascular insults (Robinson et al., 1984; Corder et al., 1993; Shi et al., 2000). Furthermore, rats’ latency, speed, error and probe function in the ZT remained unaffected by an ischemic insult in the HPC (Faraji et al., 2011a). These findings are in line with ET-1-induced ischemia in the motor cortex and striatum, showing no permanent motor impairments (Faraji et al., 2011b, 2012). These findings also suggest that the silent character of these lesions relates to their small size rather than to the location of the insults. Therefore, the present anatomical features of the hippocampal silent ischemia support the importance of the extent of the lesions, no matter which regions of the brain are involved (Kim et al., 2011).

However, our results revealed a unique behavioral feature of ET-1-induced ischemia in the dHPC when compared with tissue loss in the ventral hippocampus. Rats with ischemic lesion in the dorsal region of the hippocampus showed enhanced return behaviors during spatial investigation in the ZT that did not occur in ventral hippocampal lesions. The present data, in general, confirm previous suggestions (Vermeer et al., 2007) that although silent structural insults in the brain do not result in overt impairments, they do cause subtle behavioral deficits that may only be revealed by particularly sensitive tasks. The fact that the silent stroke is not actually silent, as reported in the present study, supports the notion to designate the term “covert” cerebral stroke as an alternative to “silent” (Longstreth et al., 2002) that may indicate a heightened risk for future infarcts with overt neurological deficits (Windham et al., 2012).

### Returns: quiet but eloquent features of spatial navigation in the ZT

A vast amount of evidence has linked the HPC to certain forms of memory (Scoville and Milner, 1957; O’Keefe and Nadel, 1979; Olton et al., 1979; Squire, 1992; Rudy and Sutherland, 1995; Eichenbaum, 2000), although different regions of the HPC support different memory functions (Moser et al., 1993, 1995; Hock and Bunsey, 1998; Bannerman et al., 1999, 2002; Pothuizen et al., 2004). The most prominent feature of the functional distribution in the hippocampus refers to the segregation of function in the dHPC and vHPC in which the rostral-dorsal regions serve spatial learning and memory function and caudal-ventral areas are involved in anxiety-related behaviors (Moser et al., 1993; Hock and Bunsey, 1998; Fanselow and Dong, 2010).

In the present study topographic disorientation was found only in rats with dorsal ischemic lesion as indicated by the enhanced return behaviors during spatial navigation. This observation confirms the concept of hippocampal functional differentiation (Moser and Moser, 1998) in which the dorsal zones of the hippocampus supports spatial information in a dynamic framework in order to locate a spatial goal in a more flexible manner.

Successful spatial navigation in the ZT is typically indicated by reduced latency and path length, but also by journeys with straight path tracks and few returns. Returns refer to act of coming back to a previously visited area during goal-directed investigation (Faraji et al., 2008). A return may represent an effort to consider a different pathway and provides the animal with operational opportunities to correct the current direction toward the spatial goal. In this perspective, each return point reflects a correction point in which the animal modifies its previous information in the context of new spatial relations created between allocentric and/or egocentric cues. The most functional outcome of the returns, therefore, is the effective spatial relation that assists in the location of a spatial goal through low error rates (not reported here), short latency, high speed and a relatively straight trajectory toward the spatial goal. The enhanced return rate found after dorsal hippocampal lesion in the absence of other deficits indicates that the animal failed to use allocentric and/or egocentric cues that normally permit recognition of previously entered routes.

Compared to sham and vHPC groups, which displayed an elevated number of the returns mainly in the first trials, the profile of elevated return frequency in dHPC rats persisted even throughout the last trials on both learning and memory days. Interestingly, dHPC rats showed the same characteristics of spatial disorientation no matter where the spatial goals were located (peripheral or central). One may argue that the enhanced return rate may be attributed to changes in sensorimotor processes (Gray and McNaughton, 1983; Vinogradova, 2001; Bast and Feldon, 2003) or potential locomotor deficits. Our data, however, indicate that changes in the number of returns may not represent locomotor sequelae of ET-1 infusion, such as hyperactivity, as revealed by the absence of group differences in speed profiles, in contrast to earlier reports of ischemia-induced hyperactivity (Plamondon et al., 2008). By contrast, based on excitatory projections from the ventral hippocampus to the nucleus accumbens that modulate sensorimotor processes, the likelihood of locomotor changes would have been greater in the vHPC group (Bardgett and Henry, 1999; Bast and Feldon, 2003). Therefore, the elevated number of returns following the dorsal hippocampal ischemia in this experiment is likely to be a cognitive deficit resulting from the dorsal hippocampal ischemia only.

## Conclusion

The present data show that despite an absence of overt spatial learning and memory impairments, silent ischemic lesions in the dHPC shift the pattern from an effective return-free investigation to an entirely return-based spatial strategy during navigation in the ZT, reflective of topographical disorientation (Bottini et al., 1990; Alsaadi et al., 2000; Marianetti et al., 2011) and impaired ability to orient and navigate in both familiar and unfamiliar surroundings (Turriziani et al., 2003; Iaria and Barton, 2010).

The findings relate to a characteristic aspect of silent ischemia-induced spatial alteration by which seemingly unaffected individuals may still show subtle deficits pertinent to the qualitative organization of behavior. The present findings propose a strategy for detection of subtle functional deficits that may be indicative of cognitive decline associated with hippocampal ischemia in a clinical population. This new rat model may serve as a clinically relevant platform to study early predictors of gradual hippocampal cell loss and cognitive impairments in silent stroke.

## Conflict of interest statement

The authors declare that the research was conducted in the absence of any commercial or financial relationships that could be construed as a potential conflict of interest.
